# COVID-19 Vaccination in a Patient With Gluten Enteropathy: A Case Report

**DOI:** 10.7759/cureus.53738

**Published:** 2024-02-06

**Authors:** Devisowmiya Thiruvengadam, Akhil Dhanesh Goel, Manoj K Gupta, Pankaj Bhardwaj, Vinoth Rajendran

**Affiliations:** 1 Community Medicine and Family Medicine, All India Institute of Medical Sciences, Jodhpur, Jodhpur, IND; 2 Community Medicine and Family Medicine, All India Institute of Medical Sciences, Gorakhpur, Gorakhpur, IND

**Keywords:** adverse reaction, gluten enteropathy, covid-19, covid-19 vaccination, covaxin

## Abstract

In India, the COVID-19 vaccination for adolescents aged 15-17 years has been started since January 2022. Gluten enteropathy, also known as celiac or nontropical sprue, can arise as an autoimmune disease of the small intestines. We report a 15-year-old female with a history of allergy to gluten-containing products who came for the first dose of the COVID-19 vaccination to adult vaccination OPD at All India Institute of Medical Sciences, Jodhpur. After taking a detailed history, she had an allergy to gluten-containing products for five years. She had no previous history of allergic reactions to injections or medicines. The first dose of Covaxin was given to this female under proper supervision, and she was followed up for any adverse events. We did not find any evidence of adverse events following the COVID-19 vaccination in people with gluten enteropathy. The patient was discharged after one hour of observation. To date, no cases of Covaxin vaccination have been reported among gluten enteropathy patients. We discuss the current evidence relating to Covaxin vaccinations, highlighting that administering the vaccine to gluten-sensitive individuals did not cause any adverse reactions. However, proper history taking and other standard procedures should be followed while administering Covaxin to any known allergies.

## Introduction

Since January 2022, when the COVID-19 vaccination for children began in India, more than 62 million teenagers have been vaccinated, as reported on the COVID-19 vaccination site. The effectiveness and safety of novel vaccines are being studied in numerous clinical trials. Still, it is challenging to predict efficacy, which is protection against severe or fatal illness [1]. Among genetically predisposed diseases, gluten enteropathy, also known as celiac or nontropical sprue, can arise as an autoimmune disease of the small intestines in genetically susceptible individuals.

The prevalence of gluten enteropathy in India is approximately 1% [2]. The effective treatment is adherence to a gluten-free diet (GFD). It is disputed if celiac disease (CD) patients have a higher risk of infection due to factors like poor nutritional state, greater intestinal permeability, and hyposplenism or if they have an inadequate immune response to vaccination [3]. Although various scientific societies have supported COVID-19 vaccines for CD, hesitation about vaccines and CD exists due to misinformation on the internet and fake news [4]. Patients with CD may experience decreased effects from the SARS-CoV-2 vaccine and may require an earlier booster vaccination [5]. With an emphasis on the COVID-19 vaccination in gluten-sensitive individuals, the present case report aims to add to the existing knowledge on CD by presenting a 15-year-old female with gluten enteropathy who had come to adult vaccination OPD for getting her first dose of Covaxin against the novel coronavirus.

## Case presentation

A 15-year-old female had come for the first dose of the COVID-19 vaccination to adult vaccination OPD at All India Institute of Medical Sciences, Jodhpur. Upon asking for a detailed history, she reported an allergy to gluten-containing products for five years. She was apparently well five years ago but subsequently experienced vomiting and diarrhea within one hour of consuming wheat-related products at a wedding function. The vomiting was of sudden onset, consisting of seven to eight episodes, nonprojectile, nonbilious, and primarily containing food particles. Following food intake, there was a history of abdominal pain but no accompanying fever, blood, or mucus discharge in the stool.

There was no reported history of difficulty breathing, lip swelling, or skin rashes. Furthermore, there was no indication of joint pain, weakness, or loss of appetite. Following the episode, she was taken to a local hospital, where she was diagnosed with gluten intolerance. The diagnosis was made clinically as per the revised criteria for the diagnosis of CD in the Report of the Working Group of the European Society for Paediatric Gastroenterology Hepatology and Nutrition (ESPGHAN). Her symptoms improved after starting a GFD.

A similar medical history was noted with her 10-year-old younger brother, who was diagnosed with a sensitivity to gluten-related products when he was two years old. No other family members reported similar complaints, and there was no history of allergy to other food products, injections, or medicines in the family. The patient had been immunized up to age without any untoward events. She had no history of any adverse events or allergies following any previous immunization. She was a vegetarian who consumed all kinds of fruits and vegetables without experiencing any discomfort.

The patient underwent an eligibility assessment for vaccination by taking a detailed history of allergies. Subsequently, she was administered Covaxin and was observed for one hour post-administration, during which no adverse events were reported. Further, the child was followed up by telephone for any adverse events for 24 hours and continues to be regularly monitored.

## Discussion

Although information on the safety and efficacy of the COVID-19 vaccine is available, there is no evidence to suggest that children with gluten enteropathy are prone to develop adverse events following the COVID-19 vaccination. In this case study, the female diagnosed with gluten enteropathy had come for the first dose of the COVID-19 vaccine.

Clinical manifestations of gluten enteropathy vary in different age groups. In infants and young children, it presents with diarrhea, abdominal distension, and failure to thrive, whereas in older children, it presents with extraintestinal manifestations like short stature, anemia, or neurologic symptoms. This child had predominantly abdominal symptoms, including abdominal discomfort, diarrhea, and vomiting [6,7]. There are different ways to confirm the diagnosis of this disease, such as a duodenal biopsy showing intraepithelial lymphocytes, villous atrophy, and a drastic response following a GFD. The diagnostic criteria of the ESPGHAN require only clinical improvement in the patient after starting a GFD.

CD is an autoimmune disease where the T cells’ own epithelial cells are destroyed in association with HLA-DQ2 or HLA-DQ8. T cells, apart from causing inflammation, also support the differentiation of B cells to produce anti-gliadin, anti-tTg, and anti-endomysial antibodies, which are used for the diagnosis and follow-up of gluten enteropathy. Due to their impact on immune system regulation, vaccines have previously been implicated as risk factors for autoimmune disorders such as CD and type 1 diabetes. However, the possible relationship between CD and vaccinations has been poorly explored [3]. CD is a disease in the mucosal lining of the small intestine that is damaged following ingestion of foods containing gluten with gliadin, which will trigger the immune system to destroy epithelium, causing it to be atrophied. The patient will then present with abdominal discomfort and diarrhea [7].

Now, in the COVID-19 pandemic, it is necessary for individuals to get vaccinated to prevent severe complications of the COVID-19 infection. This 15-year-old female had no history of allergies from previous vaccinations and was therefore considered for the COVID-19 vaccination under supervision. She was observed for 30 minutes at the vaccination site and followed up for three days, as most allergic reactions or adverse events will develop within this period. The patient did not develop allergic reactions. A study by Ibsen et al. concluded that the humoral response following SARS-CoV-2 vaccinations in CD patients is similar to that of healthy controls. Therefore, vaccinating CD patients will provide protection against SARS-CoV-2, as the antibody response is good in these populations [5]. Further, it is seen that CD patients have an increased risk for the development of infections, which includes COVID-19 infection, due to the lower levels of IgA noted in CD patients [8]. In the COVID-19 infection, a cytokine storm can occur, which is also present in CD patients, with increased expression of inflammatory cytokines. Still, a need for vaccination comes when these people are getting old and have other underlying diseases, making them vulnerable to acquiring the SARS-CoV-2 infection [9]. These findings show that increased protection is required for people with CD; hence, vaccination can be given to them.

CD was diagnosed clinically as per the revised criteria for diagnosis of CD in the Report of the Working Group of the ESPGHAN. To support the clinical diagnosis, laboratory investigations may be used to confirm the diagnosis.

## Conclusions

It is evident from this case report that Covaxin may be given to individuals with gluten enteropathy. Although there was no absolute contraindication for vaccinating a child with CD or gluten enteropathy, this child was vaccinated with Covaxin’s first dose and found no adverse events following it. The most effective way of mitigating the COVID-19 pandemic is global vaccination. However, in CD patients, the antibody production after vaccination is dynamic, requiring more investigation regarding the risk of the COVID-19 infection and vaccination response in them.
